# The latency-reversing agent HODHBt synergizes with IL-15 to enhance cytotoxic function of HIV-specific T cells

**DOI:** 10.1172/jci.insight.169028

**Published:** 2023-09-22

**Authors:** Dennis C. Copertino, Carissa S. Holmberg, Jared Weiler, Adam R. Ward, J. Natalie Howard, Callie Levinger, Alina P.S. Pang, Michael J. Corley, Friederike Dündar, Paul Zumbo, Doron Betel, Rajesh T. Gandhi, Deborah K. McMahon, Ronald J. Bosch, Noemi Linden, Bernard J. Macatangay, Joshua C. Cyktor, Joseph J. Eron, John W. Mellors, Colin Kovacs, Erika Benko, Alberto Bosque, R. Brad Jones, for the AIDS Clinical Trials Group (ACTG) A5321 Team

**Affiliations:** 1 Infectious Diseases Division, Department of Medicine, Weill Cornell Medicine, New York, New York, USA.; 2 Department of Microbiology, Immunology, and Tropical Medicine, George Washington University, Washington, DC, USA.; 3 Applied Bioinformatics Core and; 4 Department of Physiology and Biophysics, Weill Cornell Medicine, New York, New York, USA.; 5 Catenion GmbH, Berlin, Germany.; 6 Institute for Computational Biomedicine, Weill Cornell Medicine, New York, New York, USA.; 7 Division of Infectious Diseases, Massachusetts General Hospital, Boston, Massachusetts, USA.; 8 Department of Medicine, University of Pittsburgh School of Medicine, Pittsburgh, Pennsylvania, USA.; 9 Center for Biostatistics in AIDS Research, Harvard T.H. Chan School of Public Health, Boston, Massachusetts, USA.; 10 Department of Medicine, University of North Carolina at Chapel Hill, Chapel Hill, North Carolina, USA.; 11 Maple Leaf Medical Clinic, Toronto, Ontario, Canada.; 12 The ACTG A5321 Team is detailed in the Supplemental Acknowledgments.

**Keywords:** AIDS/HIV, Immunology, Adaptive immunity, Cellular immune response, Immunotherapy

## Abstract

IL-15 is under clinical investigation toward the goal of curing HIV infection because of its abilities to reverse HIV latency and enhance immune effector function. However, increased potency through combination with other agents may be needed. 3-Hydroxy-1,2,3-benzotriazin-4(3H)-one (HODHBt) enhances IL-15–mediated latency reversal and NK cell function by increasing STAT5 activation. We hypothesized that HODHBt would also synergize with IL-15, via STAT5, to directly enhance HIV-specific cytotoxic T cell responses. We showed that ex vivo IL-15 + HODHBt treatment markedly enhanced HIV-specific granzyme B–releasing T cell responses in PBMCs from antiretroviral therapy–suppressed (ART-suppressed) donors. We also observed upregulation of antigen processing and presentation in CD4^+^ T cells and increased surface MHC-I. In ex vivo PBMCs, IL-15 + HODHBt was sufficient to reduce intact proviruses in 1 of 3 ART-suppressed donors. Our findings reveal the potential for second-generation IL-15 studies incorporating HODHBt-like therapeutics. Iterative studies layering on additional latency reversal or other agents are needed to achieve consistent ex vivo reservoir reductions.

## Graphical abstract

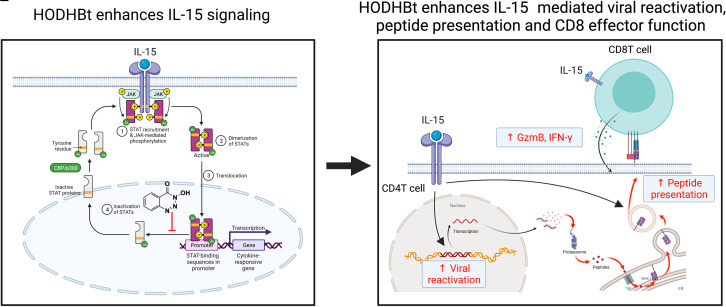

## Introduction

An estimated 37.9 million people worldwide are currently living with HIV (1). Antiretroviral therapy (ART) can arrest and reverse disease progression, as well as prevent viral transmission, but requires lifelong administration. If ART is interrupted, viral replication rapidly rebounds from reservoirs of HIV-infected cells and disease progression resumes (2–4). Extensive efforts are being directed toward the goal of curing HIV infection by enlisting the immune system to eliminate these reservoir-harboring cells, with CD8^+^ T cells comprising one promising arm of immune effectors (5, 6).

CD8^+^ T cells selectively respond to virus-infected cells by recognizing their cognate viral peptide presented in the context of MHC-I. This triggers a variety of effector functions, including the degranulation of perforin and granzymes to induce the death of infected cells (cytolysis). HIV infection typically induces a robust HIV-specific CD8^+^ T cell response, which exerts varying degrees of control on viremia (7–11). In most individuals, this is associated with a 2- to 3-log reduction in viral load from a “peak” in acute infection to a “setpoint” (7, 8). However, in rare populations of individuals termed “long-term nonprogressors” or “elite controllers,” CD8^+^ T cells are strongly implicated in achieving and maintaining suppression of viral replication to low or undetectable levels without ART (12–17). Despite this degree of efficacy in untreated infection, CD8^+^ T cell responses fail to eliminate all infected cells once viral replication is abrogated by ART.

A critical modality by which HIV reservoir-harboring cells evade elimination by CD8^+^ cytotoxic T cells (CTLs) is through viral latency, whereby a provirus can persist by maintaining a transcriptionally quiescent state and subsequently reactivating to reseed replication when ART is stopped (2–4). “Kick and kill” strategies have aimed to address this by employing latency-reversing agents (LRAs) to induce proviral expression that exposes these cells to elimination by CTLs or NK cells (18–20). Recent studies, however, have also highlighted the existence of active reservoirs of HIV with ongoing proviral transcription and translation, despite long-term ART (21–24). Indeed, HIV-specific CD8^+^ T cells show evidence of being maintained by some level of antigenic stimulation over years of ART (25), and recent observations of gradual declines in intact HIV proviruses and cell-associated HIV RNA may reflect CTL activity (26–28). However, while persisting HIV-specific CD8^+^ T cell responses can be identified by IFN-γ production, they exhibit little cytotoxic function (granzyme B release) ex vivo, and the decay of the above virologic measures is very slow (29). Thus, there is rationale to prioritize enhancing the cytotoxic character of HIV-specific CD8^+^ T cell responses — ideally alongside latency reversal — in therapeutic strategies to reduce or eliminate HIV reservoirs.

Several clinical trials have assessed LRAs either alone or in combination with therapeutic vaccines in ART-treated individuals. While some of these have shown evidence for a degree of latency reversal, appreciable reductions in HIV reservoirs have not been observed (30–34). Limitations in LRA activity very likely contributed to these outcomes, both in terms of low frequencies of reactivated proviruses and in the sense that some LRAs may induce low levels of HIV transcripts without substantive translation of HIV proteins (35, 36). Insufficient immune effector function has also been implicated. As one aspect of this, some of the LRAs used in these studies have been shown to impair CTL and NK cell function in vitro (36–38), though available evidence suggests that such impairment may not have appreciably occurred in vivo. Other agents with latency-reversing activity, however, are known to enhance cytotoxic activity of CTLs and NK cells and thus may contribute to both facets of kick and kill strategies. Prominent among these are IL-15 superagonists, such as N-803 (36), which is currently being tested in multiple clinical trials in ART-treated individuals.

Thus far, results of one clinical trial of N-803 in ART-suppressed people with HIV (PWH) have been published (39). This study showed that N-803 administration was safe, with exploratory analyses suggesting proliferation and activation of T cells and NK cells, and a small but significant decrease in levels of inducible HIV provirus. These results both call for larger studies to further investigate the impact of N-803 on HIV reservoirs and encourage the combining of N-803 with other agents with synergistic modes of action. As solo agents, the latency-reversing activities of IL-15 superagonists appear to be relatively modest, and while their enhancements of cytotoxic function in CTLs and NK cells are substantial, these effectors may need to surmount prosurvival characteristics that have been reported in HIV reservoir-harboring cells, such as overexpression of BCL-2, which antagonizes both perforin/granzyme and Fas/FasL mechanisms of CTL killing (40–42). To this end, we have previously reported that 3-hydroxy-1,2,3-benzotriazin-4(3H)-one (HODHBt) enhances signaling by IL-15 and other γc-cytokines by increasing phosphorylation and transcriptional activity of STATs upon cytokine stimulation (43, 44). HODHBt enhanced IL-2– and IL-15–mediated viral reactivation from latency in both a primary cell model of latency and cells isolated from ART-suppressed PWH in a STAT5-dependent manner (43, 44). Recently, we have shown that HODHBt also enhances IL-15–mediated effector function of NK cells (45). In the current work, we address whether HODHBt can also potentiate the ability of IL-15 to enhance the cytotoxic profiles of HIV-specific CD8^+^ T cell responses. We show that the combination of HODHBt and IL-15 treatment substantially increases granzyme B release from T cells in response to ex vivo stimulation with HIV peptides, yielding strong responses in most ART-treated donors, and assess whether this is sufficient to drive reductions in HIV reservoirs ex vivo.

## Results

**Figure 1:**
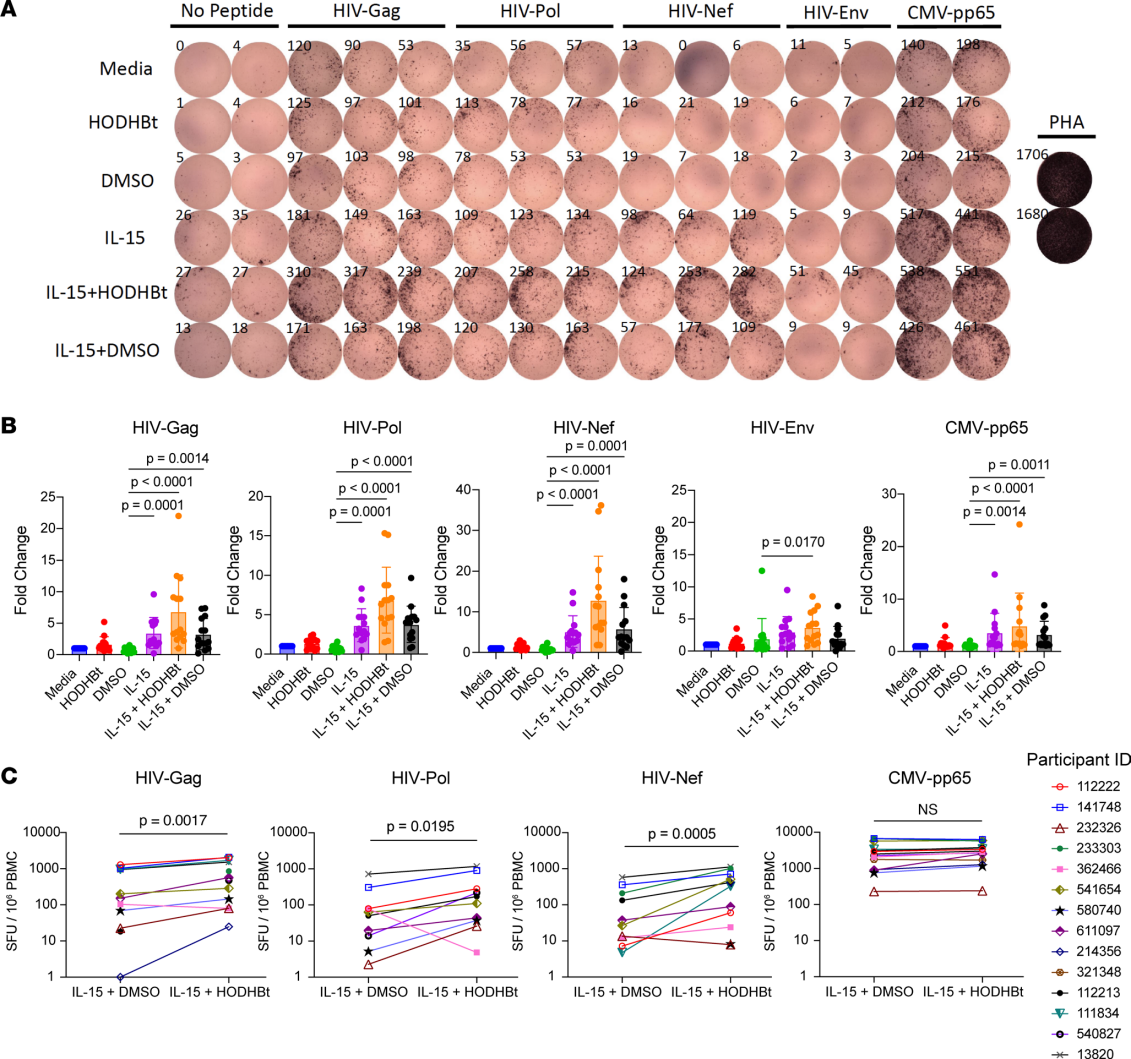
HODHBt synergizes with IL-15 to enhance HIV-specific cytotoxic CD8^+^ T cell responses ex vivo. (**A**) Representative granzyme B ELISPOT results (from 1 of 14 donors). The indicated HIV and CMV antigens were represented by overlapping peptide pools. HIV-Gag, -Pol, and -Nef stimulations were each performed in triplicate. HIV-Env peptide, CMV-pp65, no peptide, and phytohemagglutinin (PHA) were performed in duplicate. (**B** and **C**) Combined ELISPOT results from the A5321 cohort of 14 ART-treated donors. (**B**) For each peptide stimulation condition (or control), results are presented as fold-change relative to Media. Shown are means ± SD. *P* values were calculated by Friedman’s ANOVA test (1 way), with all significant *P* values displayed. (**C**) Results are from the same data set as **B**, plotted to show pairing of IL-15 + DMSO and IL-15 + HODHBt conditions across participants. Shown are SFU per million PBMCs after subtracting the background from each corresponding no-peptide treatment condition. *P* values were calculated using 2-tailed, paired, nonparametric Wilcoxon’s tests.

Using samples from the ACTG A5321 cohort, which consists of PWH with documented long-term suppression of plasma viremia on ART, we have previously demonstrated that substantial frequencies of HIV-specific T cell responses are maintained throughout years of ART (25, 29) (Supplemental Table 1; supplemental material available online with this article; https://doi.org/10.1172/jci.insight.169028DS1). While these responses are readily detectable ex vivo by IFN-γ ELISPOT, they generally exhibit very limited degranulation of granzyme B in response to peptide — consistent with weak cytotoxic activity. In the current study, we assessed whether IL-15 and HODHBt — alone or in combination — would enhance this activity in PBMCs from 14 A5321 study participants (7 men, 7 women; Supplemental Table 1). Degranulation was assessed by ELISPOT, which captures and measures granzyme B released in response to peptide stimulations. We used whole gene product pools composed of overlapping 15-mer peptides spanning HIV-Gag, HIV-Pol, HIV-Nef, HIV-Env, and CMV-pp65. Figure 1A shows a representative granzyme B ELISPOT result, using cells from a single donor, 13820. Across the cohort, we observed that IL-15 was sufficient to enhance granzyme B release in response to HIV-Gag, -Pol, and -Nef peptides but GZMB release was substantially increased by the addition of HODHBt (Figure 1B). As expected, HODHBt in the absence of IL-15 had no effect on granzyme B release. This agrees with the lack of activity of these compounds in the absence of a γc-cytokine (43–45). In directly comparing the magnitudes of granzyme B responses between IL-15 + DMSO (control) versus IL-15 + HODHBt, we observed increases from medians of Gag — 86.59 to 382.65 spot-forming units (SFU)/10^6^, *P* = 0.0017; Pol — 16.81 to 40.51 SFU/10^6^, *P* = 0.0195; and Nef — 12.81 to 356.30 SFU/10^6^, *P* = 0.0005 (each following subtraction of corresponding no-peptide conditions) (Figure 1C). CMV-pp65–specific responses, which exhibited substantial granzyme B release at baseline (Figure 1A), were not significantly enhanced by the addition of HODHBt (Figure 1C). In a subset of participants, supernatants from the ELISPOT were used to evaluate cytokine secretion using the Corplex Cytokine Panel. This panel includes IFN-γ, IL-1β, IL-4, IL-5, IL-6, IL-8, IL-10, IL-12P70, IL-22, and TNF-α. As for granzyme B, HODHBt enhanced IL-15 induction of IFN-γ but none of the other 9 cytokines analyzed (Supplemental Figure 1). Thus, HODHBt potentiates the ability of IL-15 to increase the degranulation of granzyme B and production of IFN-γ from HIV-specific T cells, ex vivo.

**Figure 2:**
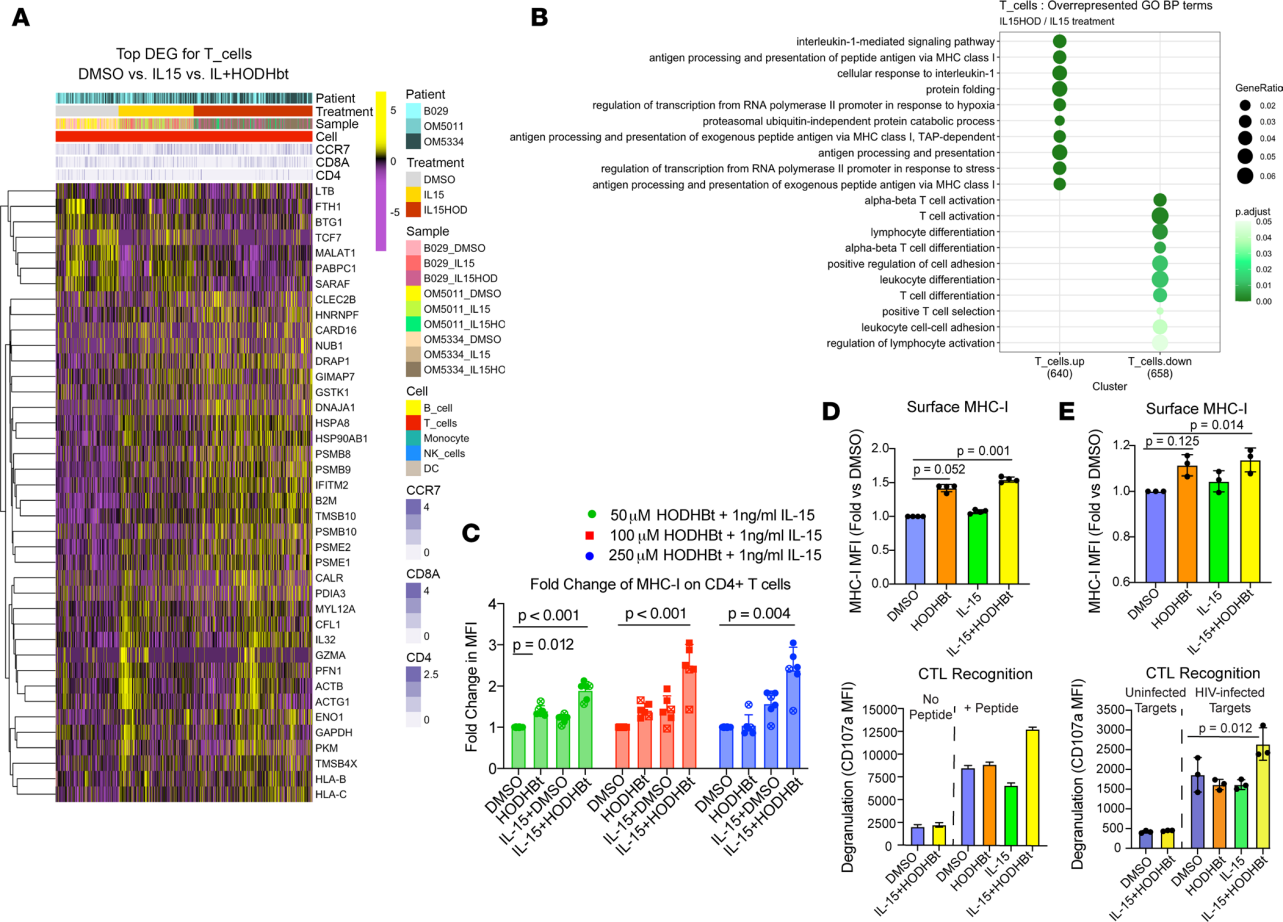
Treatment with the combination of IL-15 and HODHBt enhances surface MHC-I and antigenicity of CD4^+^ T cells. (**A** and **B**) scRNA-Seq results. (**A**) Heatmap of 40 genes that were differentially expressed (FDR < 0.05) between the treatments: DMSO (control), IL-15 + DMSO, and IL-15 + HODHBt; *n* = 3 donors. (**B**) GO terms identified by genes differentially expressed in T cells between IL-15 + DMSO and IL-15 + HODHBt conditions. *P* values were calculated by 1-sided Fisher’s exact test, and the size of each dot represents the ratio of input genes that are annotated in a term. (**C**) Flow cytometry results showing fold-changes in median fluorescence intensity (MFI) of MHC-I, relative to the DMSO control. Shown are individual data points for 6 donors, with mean ± SD. Donors with HIV are indicated with an *x* and donors without HIV as filled circles. *P* values were calculated by 1-way ANOVA with Dunnett’s multiple-comparison test. (**D**) PBMCs from ART-treated donor OM5220 were treated with DMSO, IL-15 (1 ng/mL), and HODHBt (50 μM), separately or in combination, for 4 days. CD4^+^ T cells were pulsed with a 15-mer peptide containing the RV9 epitope, then cultured or without with an autologous RV9-specific CD8^+^ T cell clone. Upper panel, surface MHC-I by treatment condition in no-clone conditions; data points indicate technical replicates. Lower panel, flow cytometry data from CD8^+^ T cell clone conditions showing the percentages of CD107a^+^ cells (degranulated). Shown are MFI ± coefficient of variation of CD107a from at least 18,000 viable CD8^+^ T cells. (**E**) CD8-depleted PBMCs from an HLA-B58^+^ donor without HIV were activated and infected with HIV_JRCSF_ or maintained as uninfected controls. After 60 hours, the antiretroviral agent T20 was added, and cells were treated with DMSO, IL-15 (20 μg/mL), and HODHBt (100 μM), separately or in combination. A CD8^+^ T cell clone specific for the HLA-B58–restricted epitope TW10 was then added to each culture across 3 replicates. Upper panel, flow cytometry data showing surface MHC-I levels. Data points indicate technical replicates and error bars represent SD. Lower panel, flow cytometry data showing the percentages of CD107a^+^CD8^+^ T cell clone. Shown are medians of technical replicates ± SD. DEG, differentially expressed gene; BP, biological process.

To more comprehensively assess the impact of HODHBt and IL-15 treatment on T cells, we performed single-cell RNA sequencing (scRNA-Seq) on PBMCs from 3 ART-treated PWH, following in vitro treatment with IL-15 + DMSO (control), IL-15 + HODHBt, or DMSO (Figure 2A). Within T cells, comparing IL-15 + DMSO and IL-15 + HODHBt scRNA-Seq revealed increased expression of genes related to Gene Ontology (GO) terms IL-1–mediated signaling, antigen processing and presentation via MHC class I, and TAP-dependent antigen processing and presentation of exogenous peptide antigen via MHC class I (Figure 2B). This analysis also revealed reductions in biological processes related to T cell activation pathways and lymphocyte and leukocyte differentiation in IL-15 + HODHBt relative to IL-15 alone (Figure 2B). The upregulation of genes involved in antigen processing and presentation led us to test and verify that MHC-I was present at higher levels on CD4^+^ T cells following treatment with IL-15 + HODHBt (Figure 2C). To test whether this would impact the antigenicity of CD4^+^ T cells — independently of HIV protein expression — we performed a recognition assay with a CD8^+^ T cell clone. Isolated primary CD4^+^ T cells were treated with 1 ng/mL IL-15 and/or 50 μM HODHBt, pulsed with a 15-mer peptide with the amino acid sequence HRLDLLLIVTRIVE, and cocultured with an autologous CD8^+^ T cell clone specific for the RV9 epitope underlined above. Recognition of target cells was assessed by exposure of CD107a, measured by flow cytometry. We observed that the combination of IL-15 + HODHBt increased recognition of target cells, mirroring increases in surface MHC-I (Figure 2D). We additionally tested whether IL-15 + HODHBt would increase the antigenicity of bona fide HIV1-infected cells. CD8-depleted PBMCs from HLA-B58^+^ donors were infected with HIV_JRCSF_, treated with 20 ng/mL IL-15 and/or 100 μM HODHBt or with a DMSO control, and cocultured with a CD8^+^ T cell clone specific for the HLA-B58–restricted TSTLQEQIGV (TW10) Gag epitope. We again observed increased degranulation in response to IL-15 + HODHBt–treated target cells, alongside a significant upregulation of MHC-I (Figure 2E). Thus, in addition to previously reported HIV latency reversal activities and enhancement of NK effector function, IL-15 + HODHBt increases the cell-intrinsic antigen presentation state of primary CD4^+^ T cells.

**Figure 3:**
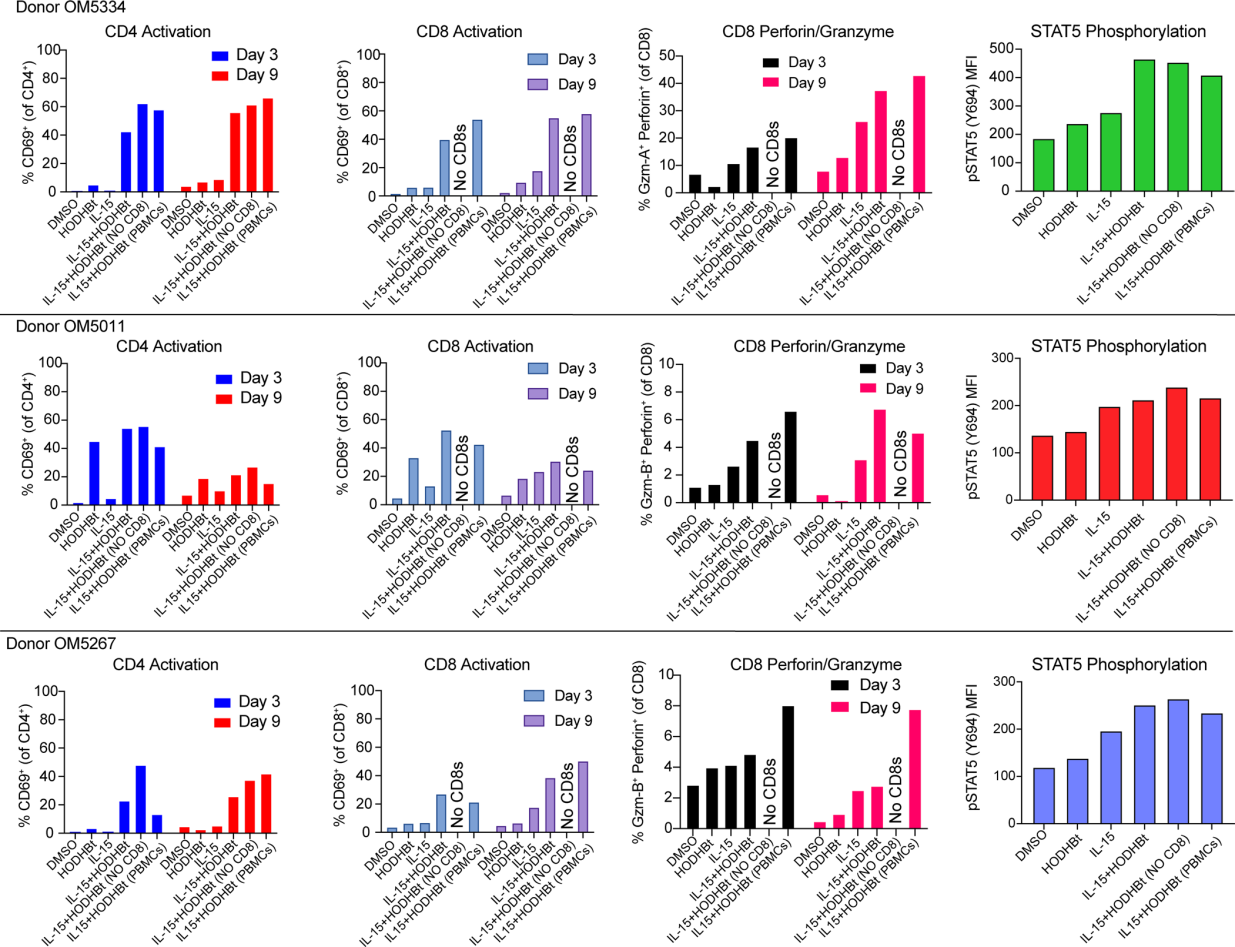
In ex vivo HIVE assays the combination of IL-15 and HODHBt induces T cell activation, STAT5 phosphorylation, and perforin/granzyme expression. Each row depicts results from a different ART-treated individual with HIV: OM5334, OM5011, and OM5267. Columns from left to right display the following flow cytometry data: i) CD4^+^ T cell activation as %CD69^+^ at days 3 and day 9 of HIVE assay, ii) CD8^+^ T cell activation as %CD69^+^ at days 3 and day 9 of HIVE assay, iii) percentage perforin and granzyme double-positive cells within CD8^+^ T cells (Note that in OM5334 granzyme A was measured, while in OM5011 and OM5267 granzyme B was measured), and iv) MFI of phosphorylated STAT5 (pSTAT5; on total PBMCs). Samples were drawn from HIVE assays at 48-hour time points.

We have previously implemented HIV eradication (HIVE) assays to assess the abilities of putative kick and kill combinations to reduce HIV reservoirs. In this past work, purified CD4^+^ T cells from ART-treated individuals were treated with LRAs and cocultured with autologous CTL clones targeting epitopes that were not escaped in reservoir virus. Here, we implemented a modified PBMC-HIVE assay to test whether the multiple favorable activities of IL-15 + HODHBt (latency reversal, increased NK and CTL cytotoxicity, and increased antigen presentation machinery) would be sufficient to reduce intact HIV proviruses. In order to include both NK and HIV-specific CD8^+^ T cells at physiological levels, IL-15 and/or HODHBt were simply added to ex vivo PBMCs and cultured for 9 days. Across each of 3 ART-treated donors, the combination of IL-15 + HODHBt drove substantial activation of CD4^+^ and CD8^+^ T cells as measured by CD69 (Figure 3). This was associated with increased expression of perforin and granzymes in total CD8^+^ T cells, as measured by flow cytometry (Figure 3 and Supplemental Figure 2. Note granzyme A was measured in OM5334 and granzyme B in donors OM5011 and OM5267. We further verified that HODHBt acted to increase STAT5 phosphorylation by IL-15 in each of the donors tested (Figure 3).

**Figure 4:**
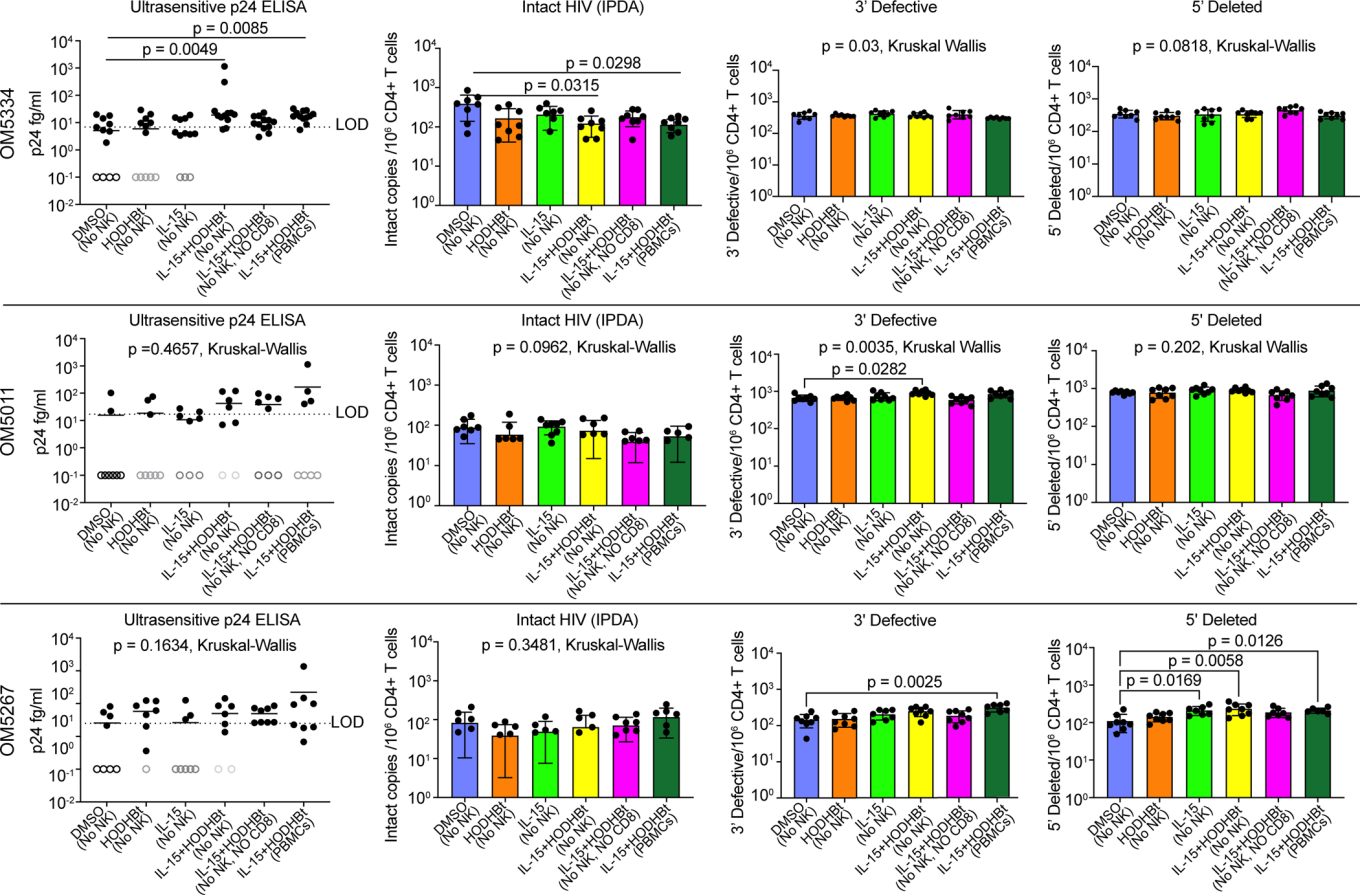
Virologic outcomes of HIVE assays. Each row depicts results from different ART-treated PWH: OM5334, OM5011, and OM5267. The leftmost columns display ultrasensitive p24 ELISA results, measured at day 3. As described in Methods, each condition was plated across multiple wells of a 96-well plate. Here, each data point corresponds to an ELISA measurement from a single well. The horizontal dashed line indicates the limit of detection (LOD). *P* values were calculated by Kruskal-Wallis test with Dunnett’s test (comparing with DMSO [no NK] condition). The remaining columns display HIV proviral DNA measurements from the intact proviral DNA assay (IPDA), from left to right: intact HIV proviruses, 3′-defective HIV proviruses (3′ deleted or hypermutated), and 5′-deleted HIV proviruses. All proviral DNA measures are presented as mean ± SD (8 replicates) copies of HIV/10^6^ CD4^+^ T cells. Kruskal-Wallis tests were performed, and resulting *P* values are shown with each graph. Where these were significant, post hoc Dunnett’s tests were performed (compared with DMSO only), and all significant *P* values are shown. A total of 8 replicates were performed for each. Means with SDs are shown.

In PBMC-HIVE assays, each condition was plated across multiple wells of a 96-deep-well plate. This serves both to enable more cell-cell contact (required for CTL and NK cell killing) than would occur in flasks, as well as to enable assessment of latency reversal in a “digital” manner, by measuring HIV-p24 protein production in each well individually. Clinical and demographic data for donors studied in these PBMC-HIVE assays are given in Supplemental Table 2. In cells from OM5334, we observed clear evidence for latency reversal in each of the IL-15 + HODHBt conditions, without such evidence for significant induction of HIV-p24 in IL-15– or HODHBt-alone conditions. To assess any impact on the HIV reservoir, we utilized the intact proviral DNA assay (IPDA), which uses a duplex droplet digital PCR strategy to distinguish the intact proviruses capable of giving rise to viral replication from the large background of defective proviruses (for example, those with deletions) that come to dominate the proviral landscapes of ART-treated individuals. For OM5334, the observed latency reversal was associated with a significant reduction in the frequency of cells harboring intact HIV proviruses (measured by IPDA) in both the whole PBMC condition (*P* = 0.0298, Figure 4) and in the condition depleted of NK cells (*P* = 0.0315) but not in the condition depleted of both NK and CD8^+^ T cells. Although there were some modest parallel trends, no significant differences between levels of 3′-defective (i.e., 3′ deleted or hypermutated) and 5′-deleted defective proviruses were observed. This is in line with expectations since many but not all defective proviruses are incapable of expressing HIV antigens (46). Neither donor OM5011 nor OM5267 showed significant latency reversal, as measured by p24 in supernatants, though a trend may be present for the IL-15 + HODHBt conditions for OM5267. Corresponding with this, we did not observe significant reductions in levels of intact HIV proviruses in either of these donors (Figure 4). Modest increases in levels of defective HIV proviruses were observed in some conditions from these donors, perhaps pointing to clonal expansion of some infected cells over this 9-day culture. Thus, our results support that a single IL-15 and HODHBt administration ex vivo can be sufficient to drive reductions in HIV reservoirs when associated with effective latency reversal measured as an increase in p24 protein expression (as observed in OM5334). This reduction in HIV reservoirs was not observed when latency reversal was not observed (OM5011 and OM5267).

## Discussion

Our results establish that HODHBt substantially increases the ability of IL-15 to induce granzyme B production and release from HIV-specific T cells. Importantly, this revealed strong cytotoxic HIV-specific responses following ex vivo stimulation with HIV peptides in individuals on long-term ART, with no pre-expansion step to increase the total frequencies of HIV-specific CD8^+^ T cells. Thus, while therapeutic vaccination to increase the magnitudes of HIV-specific CD8^+^ T cell responses represents an important strategy to boost the kill component of kick and kill interventions, our results support the use of immunomodulatory agents to enhance the functionality of existing responses as a viable alternative. Of note, the impact of IL-15 + HODHBt on HIV-specific T cell responses was more prominent and consistent than that on CMV-pp65 responses, suggesting the this combination targets a functional deficiency that is relatively specific to the former — such as either impaired differentiation or exhaustion (47–52).

A number of ongoing clinical and preclinical studies are evaluating IL-15 superagonists in the context of ART, for their abilities to either reduce HIV reservoirs or enable durable control of viremia (ClinicalTrials.gov identifiers: NCT04505501, NCT04340596, NCT05245292). Our findings suggest that if these studies show promise, compounds that share a mechanism of action with HODHBt may form the basis of next-generation combination studies. What are the prospects for HODHBt-like therapeutic agents? Given the requirement for in vitro concentrations in the range of 50–100 μM, as well as other considerations, the lead compound HODHBt itself has minimal therapeutic potential. However, in very recent mechanistic work, we have uncovered that HODHBt exerts its impact on STAT5 by inhibiting the phosphatases PTPN1 and PTPN2 (53). PTPN1 and PTPN2 have recently become attractive therapeutic targets in the context of cancer immunotherapy. Deletion of PTPN1 in immune cells enhances antitumoral immunity by increasing T cell activation, proliferation, survival, and granzyme B expression in a STAT5-dependent manner (54, 55). This increased immune effector function is not associated with the development of lymphomas, leukemia, systemic inflammation, or autoimmunity (54). Furthermore, PTPN2 deletion heightens the survival and expansion of mouse CD8^+^ T cells (56). Several new small molecules with subnanomolar activity have recently been developed to target this pathway and have shown promise in small animal models for cancer therapeutics (57). It will be important to evaluate whether these compounds also enhance latency reversal and NK and CD8^+^ effector function against HIV.

Despite the multitude of ways in which the combination of IL-15 and HODHBt perturbs PBMCs to favor reservoir elimination, we did not achieve consistent or high level reductions in HIV reservoirs ex vivo. We interpret this in light of our consistent experience, which has been that — although shocking and killing models of latency are straightforward — reductions in bona fide ex vivo reservoirs are difficult to achieve (42, 58). Having previously failed to reduce HIV reservoirs through combinations of potent LRAs and high frequencies of CTL clones, we found that we required the third component of a BCL-2 antagonist (ABT-199) to overcome pro-survival mechanisms, to achieve reservoir reductions. However, these reductions were still incomplete, variable across donors, and accompanied by a high level of ABT-199–mediated bystander toxicity. From this perspective, we view the observation that IL-15 and HODHBt were sufficient to reduce the reservoir in 1 of 3 donors as encouraging — but ultimately as pointing to a need for further enhancement (see below). While expansion to additional donors may be of interest to identify the mechanisms of donor-donor variability, this may prove very complex, especially in light of a recent study that uncovered profound heterogeneity in reservoir-harboring cells across individuals (59). We do note that the donor in whom reductions occurred both started ART earlier after infection and had a shorter duration of ART treatment than the other donors. This may be of initial interest in relation to recent suggestions that reservoirs undergo progressive immune selection on ART, with long-term ART leading to reservoirs that are both harder to reactivate and harbored in immune resistant cells (60, 61). However, extensive additional study would be needed to address this possibility. We also note that our ability to measure reservoir reductions by IPDA could have been confounded by potential proliferation of infected cells in the IL-15 + HODHBt condition. The incorporation of nearly full-length proviral sequencing in future HIVE assays will help evaluate this possibility and yield more nuanced insights into the impact of these agents on the proviral landscape.

Several approaches hold promise to enhance the consistency and impact of N-803 + HODHBt treatment on ex vivo reservoirs. Multiple rounds of treatment may be required to improve upon the observed effects, as would be expected with in vivo dosing, or additional compounds may be needed to boost either the kick or the kill, with our data pointing to room for improvement in both. With respect to the kick, significant latency reversal was only observed for OM5334 (Figure 4), and this tracked with a reduction in the reservoir in this donor. Adding LRAs with synergistic modes of action may improve the magnitude of the effect in OM5334 and unlock reductions in the other 2 donors. The pattern of p24 expression in OM5334 also points to a need for an enhanced kill. If NK and/or CD8^+^ T cells eliminated reactivated infected cells with high efficiency, then one would expect to detect p24 in the supernatants of NK- and CD8-depleted PBMCs treated with IL-15 + HODHBt but not in corresponding whole PBMCs. Many opportunities exist to enhance the kill component in this system, including the addition of i) cytopathicity-enhancing agents that counteract mechanisms of CTL resistance (such as BCL-2 antagonists), ii) antibodies to induce antibody-dependent cellular cytotoxicity against infected cells, iii) dual-affinity re-targeting antibodies or other bi-specifics to redirect CD8^+^ T cells to kill HIV-infected cells, and iv) combinations of other γc-cytokines such as IL-2 and IL-15 (62). We believe that the results presented here should motivate the testing of such combinations ex vivo but also provide impetus for parallel drug discovery efforts to identify molecules with therapeutic potential that share a mechanism of action with HODHBt.

## Methods

### Granzyme B ELISPOT assays

Mabtech Granzyme B (clone GB10, catalog 3486-2 A) ELISPOT assays against whole gene product peptides with overlap 15-mer coverage of HIV-Gag (catalog ARP-12425), HIV-Env (catalog ARP-12540), HIV-Pol (catalog ARP-12438), and HIV-Nef (catalog ARP-12545) from the NIH HIV Reagent Program and CMVpp65 peptide pool (catalog PM-PP65-2) were performed in triplicate. Multiscreen IP 96-well plates (MilliporeSigma) were coated with 7.5 μg/mL of anti–Granzyme B antibody (clone GB10, Mabtech catalog 3486-2 A) in sterile water and incubated overnight. Plates were washed, and PBMCs were added at 100,000–200,000 cells/well and stimulated with peptide pools and 0.5% DMSO and phytohemagglutinin at 2 μg/mL as negative and positive controls, respectively. Plates were incubated overnight; washed and biotinylated antibody was added (anti–Granzyme B antibody clone MT8610 from Mabtech) and incubated for 2 hours. Plates were developed with Streptavidin-ALP from Mabtech and with Color Development Buffer (Bio-Rad). Plates were washed and dried overnight and spots were counted. Responses against whole gene product peptide pools were background subtracted (thus, nonzero responses were more than 1 time background), but no other ad hoc empirical cutoff was applied — consistent with other studies examining correlations with objectively reported T cell responses as assessed by ELISPOT assay.

### Corplex cytokine panel

Cytokine secretion was assessed in the supernatants of 6 participants using the Corplex Cytokine Panel (Quanterix; 116-7BF-1-AB). This panel includes IFN-γ, IL-1β, IL-4, IL-5, IL-6, IL-8, IL-10, IL-12P70, IL-22, and TNF-α. Supernatants were thawed to room temperature, briefly vortexed, centrifuged (10,000*g*, 5 minutes, 4°C), and diluted 1:4 in sample diluent. All reagents and calibrators were prepared, and assay was performed, as per the protocol. Plates were read on the Quanterix SP-X Imaging and Analysis System.

### RNA-Seq

RNA was extracted using the AllPrep DNA/RNA Mini Kit (QIAGEN; 80204). 2-Mercaptoethanol (Bio-Rad; 1610710) was used as directed in the lysis buffer. RQ1 DNase (Promega; M6101) treatment was performed according to the manufacturer’s protocol. RNA integrity was assessed by Agilent. SMART-Seq v4 Ultra Low Input RNA plus Nextera XT DNA Sample Preparation was performed. The DNA library preparation, quality control, and sequencing were all performed by the Genomics Resources Core Facility at Weill Cornell Medicine. Illumina NovaSeq 6000 was used for sequencing, using an S2 flow cell and paired-end 2 × 50 cycles.

#### 5′ ScRNA-Seq library preparation and sequencing.

Following in vitro treatment with IL-15 + DMSO (control), IL-15 + HODHBt (both from R&D Systems), or DMSO, PBMCs from 3 ARV-suppressed PWH were resuspended at a density of 1,000 cells/μL in PBS plus 0.04% bovine serum albumin on ice and loaded into the 10x Genomics Chromium Controller with a target capture of about 5,000 cells per condition/donor using the single-cell immune profiling 5′ chip and reagent/gel bead kits according to the manufacturer’s protocol. Barcoded sample libraries were quantified and pooled using Qubit fluorometric quantification (Thermo Fisher Scientific) and Bioanalyzer (Agilent). Libraries were sequenced on an Illumina NovaSeq in a 26 × 8 × 91 bp configuration.

#### 5′ ScRNA-Seq data analysis.

FASTQ files were processed using Cell Ranger 6.1.1 and mapped to a custom combined reference with HXB2 HIV reference genome added to human GRCh38 reference FASTA and GTF files. Following the workflow described by Amezquita et al., scRNA-Seq analysis was carried out with R/Bioconductor packages (63). Quality control was carried out for each sample separately with functions from the scuttle package v.1.4.0 (64), and cells with low gene content (below 10 × 10^–2.5^ to 10 × 10^–2.75^, depending on the sample) and high mitochondrial gene content (>3 median absolute deviations; default scuttle setting) were removed from further analyses. Genes with zero expression across all cells were removed from the matrix. Cell cycle scores were calculated with the scuttle:cyclone() function using human cell cycle marker genes provided by scuttle. The different count matrices across all samples were then scaled for sequencing depth differences and log-transformed using multiBatchNorm from the batchelor v.1.10.0 (65, 66) package. Cell types were annotated with SingleR v. 1.8.1 using celldex:HumanPrimaryCellAtlasData() (67). Differentially expressed genes (FDR < 0.05) were detected using the pseudoBulkDGE function from the scran package (68), which uses the quasi-likelihood method implemented by edgeR (69). GO analysis was performed using the enrichGO function from clusterProfiler v. 3.10.1 (70). Code and processed data can be found at https://github.com/abcwcm/CopertinoHODHbt (commit ID e4b5fbe). We have provided the least processed data that do not contain the actual genetic sequencing information from study participants (which is Protected Health Information).

#### RNA-Seq validation.

A total of 20 × 10^6^ PBMCs from 2 PWH and 4 previously cryopreserved leuko-paks were thawed, washed with warm R-10 medium (RPMI 1640 [Thermo Fisher Scientific] supplemented with 10% FBS, l-glutamine, 10 mM HEPES, and penicillin/streptomycin) twice, and placed at a concentration of 4 × 10^6^ cells/mL at 37°C and 5% CO_2_ in 2 mL per condition with 12 conditions in a 12-well plate. PBMCs from each participant were treated for 4 days with increasing amounts of DMSO, HODHBt (50 μM, or 100 μM, or 150 μM), IL-15 (1 ng/mL, or 10 ng/mL, or 25 ng/mL), or the combination of HODHBt with IL-15 (50 μM, or 100 μM, or 150 μM + 1 ng/mL, 10 ng/mL, or 25 ng/mL, respectively). Flow cytometry was performed at day 1 and day 4 after adding stimuli, using the following: fixable viability dye (aqua; Thermo Fisher Scientific, catalog L34966); anti-human CD3 (clone SK7; BioLegend, catalog 344842); anti-human CD4 (clone A161 A1; BioLegend, catalog 357418); anti-human CD8 (clone RPA-T8; BioLegend, catalog 301040); anti-human CD56 (clone HCD56, catalog 318336) anti–human HLA-A,B,C (clone W6/32, catalog 311426); anti-human perforin (clone dG9; BioLegend, catalog 308126); and anti-human granzyme B (clone GB11; BioLegend, catalog 515403). Cytofix/Cytoperm (BD Biosciences; 554722) was used to fix and permeabilize cells, and 1× Perm Wash buffer was used to dilute intracellular antibodies.

### Ex vivo HIVE assay

Cryopreserved PBMCs were thawed and washed twice with warm R-10 medium (RPMI 1640 [Thermo Fisher Scientific] supplemented with 10% FBS, l-glutamine, 10 mM HEPES, and penicillin/streptomycin). EasySep Human CD56 Positive Selection Kit II (catalog 17855) and EasySep Human CD8 Positive Selection Kit II (catalog 17853) selections were performed according to manufacturer’s instructions (STEMCELL Technologies). Cells were then resuspended at 2 × 10^6^ cells/mL, in R-10 medium with 10 μM T20 (NIH, catalog ARP-12732), 1 μM tenofovir (NIH, catalog ARP-10199), 1 μM emtricitabine (NIH, catalog ARP-10071), 1 μM nevirapine (NIH, catalog ARP-4666), and 10 U/mL human DNase I (ProSpec, catalog ENZ-319-c). LRAs — IL-15 and/or HODHBt — or DMSO (Corning, catalog 25-950-CQC) were added accordingly, and cells were incubated for 9 days at 37°C at 5% CO_2_ in a deep-well plate (Thermo Fisher Scientific, catalog 278743). A total of 10 μL of cells from 12 wells were removed and combined for staining at day 2 to assess p–STAT5-Y694 (see below). LRAs included in certain conditions IL-15 1 ng/mL (NIH National Cancer Institute, catalog 50341) and HODHBt 50 μM (Tocris, catalog 6994). The media were changed on days 3 and 6, and supernatants were clarified of debris at 1,000*g* for 10 minutes and stored at –80°C, until being analyzed by ultrasensitive p24 (see below). Cells were stained for flow cytometry to assess viability and activation at days 3, 6, and 9. A total of 10 μL of resuspended cells were removed from 12 wells and stained for flow cytometry. On day 9 all cells were resuspended and removed, and CD4^+^ T cells were enriched from PBMCs using the EasySep Human CD4+ T Cell Isolation Kit (catalog 19052). DNA and RNA were co-extracted using the AllPrep Mini (catalog 80204) from the same CD4^+^ cell sample. RNA was used for RNA-Seq (see RNA-Seq section).

#### Flow cytometry.

At each time point sampled, cells were stained with 1:100 dilutions of the antibodies in PBS with 2% FBS, 2 mM EDTA, fixable viability dye (aqua; Thermo Fisher Scientific, catalog L34966), anti-human CD3 (clone SK7; BioLegend, catalog 344842), anti-human CD4 (clone A161 A1; BioLegend, catalog 357418), anti-human CD8 (clone RPA-T8; BioLegend, catalog 301040), anti-human CD69 (clone FN50; Invitrogen, catalog 47-0699-42), anti-human GZMA (clone CB9; BioLegend, catalog 507215), anti-human GZMB (clone GB11; BioLegend, catalog 515403), anti-human HLA-DR (clone LN3, BioLegend, catalog 327002), anti-human perforin (clone dG9; BioLegend, catalog 308126), and anti-Bcl2 (clone 100; BioLegend, catalog 658709). Cytofix/Cytoperm (BD Biosciences, catalog 554722) was used to fix and permeabilize cells, and 1× Perm Wash buffer was used to dilute intracellular antibodies.

#### p-STAT5 (Y694) staining.

To analyze p-STAT5, 100,000 cells were first stained with a viability dye (Fixable Viability Dye eFluor 450, Affymetrix, Thermo Fisher Scientific) for 10 minutes at 4°C. Cells were then fixed with 100 μL of prewarmed (37°C) Fix Buffer I (Becton Dickinson) for 10 minutes at 37°C. Cells were washed once with 1 mL of PBS + 3% FBS. Cells were then permeabilized while vortexing with 100 μL of Perm Buffer III (Becton Dickinson) and incubated for 30 minutes on ice. Cells were washed once with 1 mL of PBS + 3% FBS and stained with 2 μL of p-STAT5–Alexa Fluor 488 (clone 47/Stat5 [pY694]; Becton Dickinson, catalog 612598) in 100 μL of PBS + 3% FBS for 1 hour at room temperature in the dark. Finally, cells were washed once with 1 mL of PBS + 3% FBS, and p-STAT5 was measured by flow cytometric analysis in a FACSCanto II flow cytometer using the FACSDiva software (Becton Dickinson) and analyzed using FlowJo (Tree Star Inc).

#### Ultrasensitive p24 ELISA.

Supernatants were collected for p24 detection using an ultrasensitive ELISA (SP-X technology) as described by Levinger et al. (71). As treatment conditions of the HIVE assays were plated across multiple wells of 96-well plates, multiple independent supernatant measures were made for each. Briefly, plates coated with 1 μg/mL capture antibody (Capricorn Products, catalog HIV-018-48303) were incubated with 50 μL of each culture condition for 2 hours on a shaker. Prior to analysis, viruses were inactivated by adding 1% Triton X-100 (Thermo Fisher Scientific). Plates were washed 4 times and patted dry to remove excess wash buffer (ELISA wash buffer, Quanterix). Before addition of the biotinylated anti-p24 detection antibody (1 μg/mL, clone: 39/5.4A, ZeptoMetrix Corporation, catalog 0801080), a blockade step was performed by using 5% nonfat milk for 30 minutes. Detection of p24 was performed by incubation with streptavidin-HRP (Quanterix) for 30 minutes on a shaker. The plate was washed 6 times and patted dry after each step. After adding mixed 25 μL Stable Peroxide (Quanterix) and 25 μL SuperSignal Luminol (Quanterix) for each well, the plate was immediately read on the SP-X Imager (Quanterix). P24 was calculated based on a standard curve using recombinant p24 protein (ZeptoMetrix Corporation, catalog 0801002) prepared in the same media at concentrations 100, 20, 4, 0.8, 0.16, 0.032, 0.0064, and 0 pg/mL.

### Peptide-pulsed degranulation assay

PBMCs from OM5220 were incubated with DMSO 0.05% (Corning, catalog 25-950-CQC), HODHBt (50 μM), IL-15 (1 ng/mL) with DMSO 0.05%, or the combination of HODHBt (50 μM) with IL-15 (1 ng/mL) for 4 days. Cells were washed with MACS and CD4+ T cells Enriched (STEMCELL Technologies, EasySep, catalog 19052). CD4^+^ T cells were then stained with CellTrace Far Red 0.05 μM (Invitrogen, catalog C34564), washed once, and pulsed with Env192 HRLDLLLIVTRIVE (NIH-ARP, catalog 9480), then washed 4 times. The CTL RV9 was stained with CellTrace Violet 0.5 μM (Invitrogen, catalog C34557) and washed 4 times. CD4^+^ T cells were placed at a concentration of 100,000 cells/well in each well, and CTLs were added at 1:1 effector-to-target ratios with the same drugs they had been treated with for the previous 4 days. A 1:200 concentration of PE–anti-CD107a (LAMP-1 antibody clone H4A3; BioLegend, catalog 328608) was added to each well and incubated for 5 hours in a round-bottom, 96-well plate (Corning, catalog 08-772-17). Cells were centrifuged (400*g*, 5 minutes, at room temperature), and supernatants were saved at –80°C for further analysis. T cells were washed in MACS buffer (2% FBS [Thermo Fisher Scientific, catalog 16-140-071], 2 mM EDTA [Invitrogen, catalog AM9260G]), in Dulbecco’s PBS (Gibco, catalog 14190-136), and surface stained with fixable viability dye (aqua; Thermo Fisher Scientific, catalog L34966) and the following fluorochrome-conjugated antibodies: anti-human CD3 (clone SK7; BioLegend, catalog 344842), anti-human CD4 (clone A161 A1; BioLegend, catalog 357416), anti-human CD8 (clone RPA-T8; BioLegend, catalog 301040), and anti-human HLA-A,B,C (clone W6/32; BioLegend, catalog 311430). All antibodies were added at a concentration of 1:100. Cells were fixed using 4% paraformaldehyde and analyzed on an Attune NxT Flow Cytometer (Thermo Fisher Scientific) immediately. Data were analyzed using FlowJo software.

### Infected cell degranulation assay

PBMCs from deidentified HLA-B58^+^ HIV-negative donors (AllCells) were depleted of CD8^+^ T cells (STEMCELL Technologies, EasySep, catalog 17853) and activated with ImmunoCult Human CD3/CD28 T Cell Activator (STEMCELL Technologies, catalog 10971) (for 72 hours in R-10 medium with 50 U/mL IL-2 [National Cancer Institute BRB Preclinical Biologics Repository]) (R-10-50). Two-thirds of these cells were then infected with HIV_JRCSF_ at an MOI of 0.1 while the other third was maintained as an uninfected control. After 60 hours of culture, cells were washed and resuspended in R-10-50 with 10 μM T20 (NIH, catalog ARP-12732). Infected cells were divided into 4 groups and treated with i) 20 ng/mL IL-15, ii) 100 μM HODHBt, iii) 20 ng/mL IL-15 + 100 μM HODHBt, or iv) 0.1% DMSO (matched to concentration in 100 μM HODHBt). Uninfected cells were divided into 2 groups and treated with either i) 20 ng/mL IL-15 + 100 μM HODHBt or ii) 0.1% DMSO. After 13 hours of treatment, cells were cocultured for 8 hours with a CD8^+^ T cell clone specific for the Gag TW10 epitope in R-10-50 with 1:100 APC–anti-CD107a (LAMP-1 antibody clone H4A3; BioLegend, catalog 328619). Cells were then stained with fixable viability dye (aqua; Thermo Fisher Scientific, catalog L34966) and the following fluorochrome-conjugated antibodies: anti-human CD3 (clone SK7; BioLegend, catalog 344842), anti-human CD4 (clone RPA-T4; BioLegend, catalog 300526), anti-human CD8 (clone RPA-T8; BioLegend, catalog 301040), and anti-human HLA-A,B,C (clone W6/32; BioLegend, catalog 311440). All antibodies were added at a concentration of 1:100. Cells were fixed using 4% paraformaldehyde and analyzed on an Attune NxT Flow Cytometer. Data were analyzed using FlowJo software.

### Duplex digital droplet PCR (IPDA)

Genomic DNA was isolated from CD4^+^ T cells using the AllPrep DNA/RNA Mini Kit with precautions to minimize DNA shearing. Intact HIV copies/million CD4^+^ T cells were determined by droplet digital PCR (ddPCR) using the IPDA, where HIV and human RPP30 reactions were conducted independently in parallel and copies were normalized to the quantity of input DNA. In each ddPCR reaction, a median of 7.5 ng for RPP30, or a median of 708.75 ng for HIV (304.5–750 ng) of genomic DNA was combined with ddPCR Supermix for Probes (No dUTPs) (Bio-Rad, catalog 1863010), primers (final concentration 900 nM, Integrated DNA Technologies), probe(s) (final concentration 250 nM, Thermo Fisher Scientific), and nuclease-free water. Primer and probe sequences (5′ to 3′) were RPP30 Forward Primer GATTTGGACCTGCGAGCG, RPP30 Probe VIC CTGACCTGAAGGCTCT-MGBNFQ, RPP30 Reverse Primer GCGGCTGTCTCCACAAGT; RPP30 Shear Forward Primer CCATTTGCTGCTCCTTGGG, RPP30 Shear Probe FAM-AAGGAGCAAGGTTCTATTGTAG-MGBNFQ, RPP30 Shear Reverse Primer CATGCAAAGGAGGAAGCCG; HIV Ψ Forward Primer CAGGACTCGGCTTGCTGAAG, HIV Ψ Probe FAM-TTTTGGCGTACTCACCAGT-MGBNFQ, HIV Ψ Reverse Primer GCACCCATCTCTCTCCTTCTAGC; and HIV env Forward Primer AGTGGTGCAGAGAGAAAAAAGAGC, HIV env Probe VIC-CCTTGGGTTCTTGGGA-MGBNFQ, anti-Hypermutant env Probe CCTTAGGTTCTTAGGAGC-MGBNFQ, HIV env Reverse Primer GTCTGGCCTGTACCGTCAGC; secondary *env* Forward Primer ACTATGGGCGCAGCGTC. In OM5334, the above env primer/probes did not yield amplification — likely due to viral sequence diversity (72). We therefore used the following secondary primer/probes: secondary *env* Probe VIC-CTGGCCTGTACCGTCAG-MGBNFQ and Secondary *env* Reverse Primer CCCCAGACTGTGAGTTGCA. Droplets were prepared using the QX200 Droplet Generator (Bio-Rad) and cycled at 95°C for 10 minutes; 45 cycles of (94°C for 30 seconds, 59°C for 1 minute) and 98°C for 10 minutes. Droplets were analyzed on a QX200 Droplet Reader (Bio-Rad) using QuantaSoft software (Bio-Rad, version 1.7.4). Between 4 and 8 technical replicates were performed for each participant sample. Intact HIV copies (Ψ and env-RRE double-positive droplets) were corrected for DNA shearing based on the frequency of RPP30 and RPP30-Shear double-positive droplets. As in our previous studies, outliers were defined as replicates that deviated from the mean by more than 2 times the SD and were excluded.

### Statistics

Statistical analyses were performed in Prism GraphPad. Statistical tests used are indicated in the corresponding figure legends. *P* values less than 0.05 were considered significant.

### Study approval

Each ACTG A5321 clinical research site had the A5321 protocol and consent form, and its relevant parental protocols and consent forms, approved by their local IRBs, as well as registered with and approved by the NIH Division of AIDS Regulatory Support Center Protocol Registration Office (Bethesda, Maryland, USA), prior to any participant recruitment and enrollment. Once a participant for study entry was identified, details were carefully discussed with the prospective participant by clinical staff at the site. The participant (or, when necessary, the parent or legal guardian if the participant was under guardianship) was asked to read and sign the ACTG-approved protocol consent form. Samples from the Maple Leaf Clinic were collected through a protocol approved by the University of Toronto Institutional Review Board. Secondary use of these was approved by the Weill Cornell Institutional Review Board. All participants were adults and gave written informed consent.

### Data availability

Values for data points shown in graphs are provided in the Supporting Data Values file. For RNA-Seq, our code and processed data can be found at https://github.com/abcwcm/CopertinoHODHbt (commit ID e4b5fbe).

## Author contributions

DCC, CSH, ARW, JW, FD, PZ, DB, AB, and RBJ conceived, designed, and analyzed the experiments. RTG, DKM, RJB, BJM, JCC, JJE, JWM, CK, and EB designed and executed clinical protocols. DCC, CSH, ARW, JNH, CL, APSP, MJC, NL, and JW carried out the experiments. RBJ wrote the manuscript with input from all coauthors.

## Supplementary Material

Supplemental data

Supporting data values

## Version 1 08/15/2023

In-Press Preview

## Version 2 09/22/2023

Electronic publication

## References

[1] https://www.unaids.org/sites/default/files/media_asset/2019-UNAIDS-data_en.pdf.

[2] Chun TW (1997). Presence of an inducible HIV-1 latent reservoir during highly active antiretroviral therapy. Proc Natl Acad Sci U S A.

[3] Wong JK (1997). Recovery of replication-competent HIV despite prolonged suppression of plasma viremia. Science.

[4] Finzi D (1997). Identification of a reservoir for HIV-1 in patients on highly active antiretroviral therapy. Science.

[5] Jones RB, Walker BD (2016). HIV-specific CD8^+^ T cells and HIV eradication. J Clin Invest.

[6] Deeks SG (2021). Research priorities for an HIV cure: International AIDS Society Global Scientific Strategy 2021. Nat Med.

[7] Borrow P (1994). Virus-specific CD8+ cytotoxic T-lymphocyte activity associated with control of viremia in primary human immunodeficiency virus type 1 infection. J Virol.

[8] Koup RA (1994). Temporal association of cellular immune responses with the initial control of viremia in primary human immunodeficiency virus type 1 syndrome. J Virol.

[9] Jin X (1999). Dramatic rise in plasma viremia after CD8(+) T cell depletion in simian immunodeficiency virus-infected macaques. J Exp Med.

[10] Schmitz JE (1999). Control of viremia in simian immunodeficiency virus infection by CD8+ lymphocytes. Science.

[11] Walker B, McMichael A (2012). The T-cell response to HIV. Cold Spring Harb Perspect Med.

[12] Yang OO (2017). Demographics and natural history of HIV-1-infected spontaneous controllers of viremia. AIDS.

[13] Migueles SA (2000). HLA B*5701 is highly associated with restriction of virus replication in a subgroup of HIV-infected long term nonprogressors. Proc Natl Acad Sci U S A.

[14] International HIVCS (2010). The major genetic determinants of HIV-1 control affect HLA class I peptide presentation. Science.

[15] Kaslow RA (1996). Influence of combinations of human major histocompatibility complex genes on the course of HIV-1 infection. Nat Med.

[16] Migueles SA (2002). HIV-specific CD8^+^ T cell proliferation is coupled to perforin expression and is maintained in nonprogressors. Nat Immunol.

[17] Ndhlovu ZM (2015). The breadth of expandable memory CD8^+^ T cells inversely correlates with residual viral loads in HIV elite controllers. J Virol.

[18] Deeks SG (2012). HIV: shock and kill. Nature.

[19] Shan L (2012). Stimulation of HIV-1-specific cytolytic T lymphocytes facilitates elimination of latent viral reservoir after virus reactivation. Immunity.

[20] Nixon CC (2020). Systemic HIV and SIV latency reversal via non-canonical NF-κB signalling in vivo. Nature.

[21] Wiegand A (2017). Single-cell analysis of HIV-1 transcriptional activity reveals expression of proviruses in expanded clones during ART. Proc Natl Acad Sci U S A.

[22] Wu G (2021). Gag p24 is a marker of human immunodeficiency virus expression in tissues and correlates with immune response. J Infect Dis.

[23] DeMaster LK (2015). A subset of CD4/CD8 double-negative T cells expresses HIV proteins in patients on antiretroviral therapy. J Virol.

[24] McManus WR (2019). HIV-1 in lymph nodes is maintained by cellular proliferation during antiretroviral therapy. J Clin Invest.

[25] Thomas AS (2017). T-cell responses targeting HIV Nef uniquely correlate with infected cell frequencies after long-term antiretroviral therapy. PLoS Pathog.

[26] Peluso MJ (2020). Differential decay of intact and defective proviral DNA in HIV-1-infected individuals on suppressive antiretroviral therapy. JCI Insight.

[27] Cho A (2022). Longitudinal clonal dynamics of HIV-1 latent reservoirs measured by combination quadruplex polymerase chain reaction and sequencing. Proc Natl Acad Sci U S A.

[28] Gandhi RT (2021). Selective decay of intact HIV-1 proviral DNA on antiretroviral therapy. J Infect Dis.

[29] Stevenson EM (2021). HIV-specific T cell responses reflect substantive in vivo interactions with antigen despite long-term therapy. JCI Insight.

[30] Spivak AM, Planelles V (2016). HIV-1 eradication: early trials (and tribulations). Trends Mol Med.

[31] Van Lint C (2013). HIV-1 transcription and latency: an update. Retrovirology.

[32] Sogaard OS (2015). The depsipeptide romidepsin reverses HIV-1 latency in vivo. PLoS Pathog.

[33] Rasmussen TA (2014). Panobinostat, a histone deacetylase inhibitor, for latent-virus reactivation in HIV-infected patients on suppressive antiretroviral therapy: a phase 1/2, single group, clinical trial. Lancet HIV.

[34] Fidler S (2020). Antiretroviral therapy alone versus antiretroviral therapy with a kick and kill approach, on measures of the HIV reservoir in participants with recent HIV infection (the RIVER trial): a phase 2, randomised trial. Lancet.

[35] Grau-Exposito J (2019). Latency reversal agents affect differently the latent reservoir present in distinct CD4^+^ T subpopulations. PLoS Pathog.

[36] Jones RB (2016). A subset of latency-reversing agents expose HIV-infected resting CD4^+^ T-cells to recognition by cytotoxic T-lymphocytes. PLoS Pathog.

[37] Walker-Sperling VE (2016). The effect of latency reversal agents on primary CD8^+^ T cells: implications for shock and kill strategies for human immunodeficiency virus eradication. EBioMedicine.

[38] Clutton G (2016). The differential short- and long-term effects of HIV-1 latency-reversing agents on T cell function. Sci Rep.

[39] Miller JS (2022). Safety and virologic impact of the IL-15 superagonist N-803 in people living with HIV: a phase 1 trial. Nat Med.

[40] Sutton VR (1997). Bcl-2 prevents apoptosis induced by perforin and granzyme B, but not that mediated by whole cytotoxic lymphocytes. J Immunol.

[41] Lickliter JD (2007). Small-molecule Bcl-2 inhibitors sensitise tumour cells to immune-mediated destruction. Br J Cancer.

[42] Ren Y (2020). BCL-2 antagonism sensitizes cytotoxic T cell-resistant HIV reservoirs to elimination ex vivo. J Clin Invest.

[43] Bosque A (2017). Benzotriazoles reactivate latent HIV-1 through inactivation of STAT5 SUMOylation. Cell Rep.

[44] Sorensen ES (2020). Structure-activity relationship analysis of benzotriazine analogues as HIV-1 latency-reversing agents. Antimicrob Agents Chemother.

[45] Macedo AB (2022). The HIV latency reversal agent HODHBt enhances NK cell effector and memory-like functions by increasing interleukin-15-mediated STAT activation. J Virol.

[46] Pollack RA (2017). Defective HIV-1 proviruses are expressed and can be recognized by cytotoxic T lymphocytes, which shape the proviral landscape. Cell Host Microbe.

[47] Champagne P (2001). Skewed maturation of memory HIV-specific CD8 T lymphocytes. Nature.

[48] Addo MM (2007). Fully differentiated HIV-1 specific CD8^+^ T effector cells are more frequently detectable in controlled than in progressive HIV-1 infection. PLoS One.

[49] Trautmann L (2006). Upregulation of PD-1 expression on HIV-specific CD8^+^ T cells leads to reversible immune dysfunction. Nat Med.

[50] Day CL (2006). PD-1 expression on HIV-specific T cells is associated with T-cell exhaustion and disease progression. Nature.

[51] Petrovas C (2006). PD-1 is a regulator of virus-specific CD8^+^ T cell survival in HIV infection. J Exp Med.

[52] Jones RB (2008). Tim-3 expression defines a novel population of dysfunctional T cells with highly elevated frequencies in progressive HIV-1 infection. J Exp Med.

[54] Wiede F (2022). PTP1B is an intracellular checkpoint that limits T-cell and CAR T-cell antitumor immunity. Cancer Discov.

[55] Bentires-Alj M, Neel BG (2007). Protein-tyrosine phosphatase 1B is required for HER2/Neu-induced breast cancer. Cancer Res.

[56] Flosbach M (2020). PTPN2 deficiency enhances programmed T cell expansion and survival capacity of activated T cells. Cell Rep.

[57] Liang S (2023). A small molecule inhibitor of PTP1B and PTPN2 enhances T cell anti-tumor immunity. Nat Commun.

[58] Huang SH (2018). Latent HIV reservoirs exhibit inherent resistance to elimination by CD8^+^ T cells. J Clin Invest.

[59] Wu VH (2023). Profound phenotypic and epigenetic heterogeneity of the HIV-1-infected CD4^+^ T cell reservoir. Nat Immunol.

[60] Sun W (2023). Phenotypic signatures of immune selection in HIV-1 reservoir cells. Nature.

[61] Huang SH (2019). Have cells harboring the HIV reservoir been immunoedited?. Front Immunol.

[62] Ward AR (2021). Immunological approaches to HIV cure. Semin Immunol.

[63] Amezquita RA (2020). Publisher correction: orchestrating single-cell analysis with Bioconductor. Nat Methods.

[64] McCarthy DJ (2017). Scater: pre-processing, quality control, normalization and visualization of single-cell RNA-seq data in R. Bioinformatics.

[65] Lun AT (2016). Pooling across cells to normalize single-cell RNA sequencing data with many zero counts. Genome Biol.

[66] https://marionilab.github.io/FurtherMNN2018/.

[67] Aran D (2019). Reference-based analysis of lung single-cell sequencing reveals a transitional profibrotic macrophage. Nat Immunol.

[68] Lun AT (2016). A step-by-step workflow for low-level analysis of single-cell RNA-seq data with Bioconductor. F1000Res.

[69] Robinson MD (2010). edgeR: a Bioconductor package for differential expression analysis of digital gene expression data. Bioinformatics.

[70] Yu G (2012). clusterProfiler: an R package for comparing biological themes among gene clusters. OMICS.

[71] Levinger C (2021). An ultrasensitive planar array p24 Gag ELISA to detect HIV-1 in diverse biological matrixes. Sci Rep.

[72] Kinloch NN (2021). HIV-1 diversity considerations in the application of the Intact Proviral DNA Assay (IPDA). Nat Commun.

