# Supplementary material for: The latency-reversing agent HODHBt synergizes with IL-15 to enhance cytotoxic function of HIV-specific T cells
Source: JCI Insight. 2023 Sep 22;8(18):e169028. doi: 10.1172/jci.insight.169028 (PMC10561764; doi:10.1172/jci.insight.169028)
Supplement: Supplemental data [file jciinsight-8-169028-s005.pdf]

Supplemental Data for

"The latency reversing agent HODHBt synergizes with IL-15 to enhance cytotoxic function of HIV-specific T-cells", by Copertino *et al*

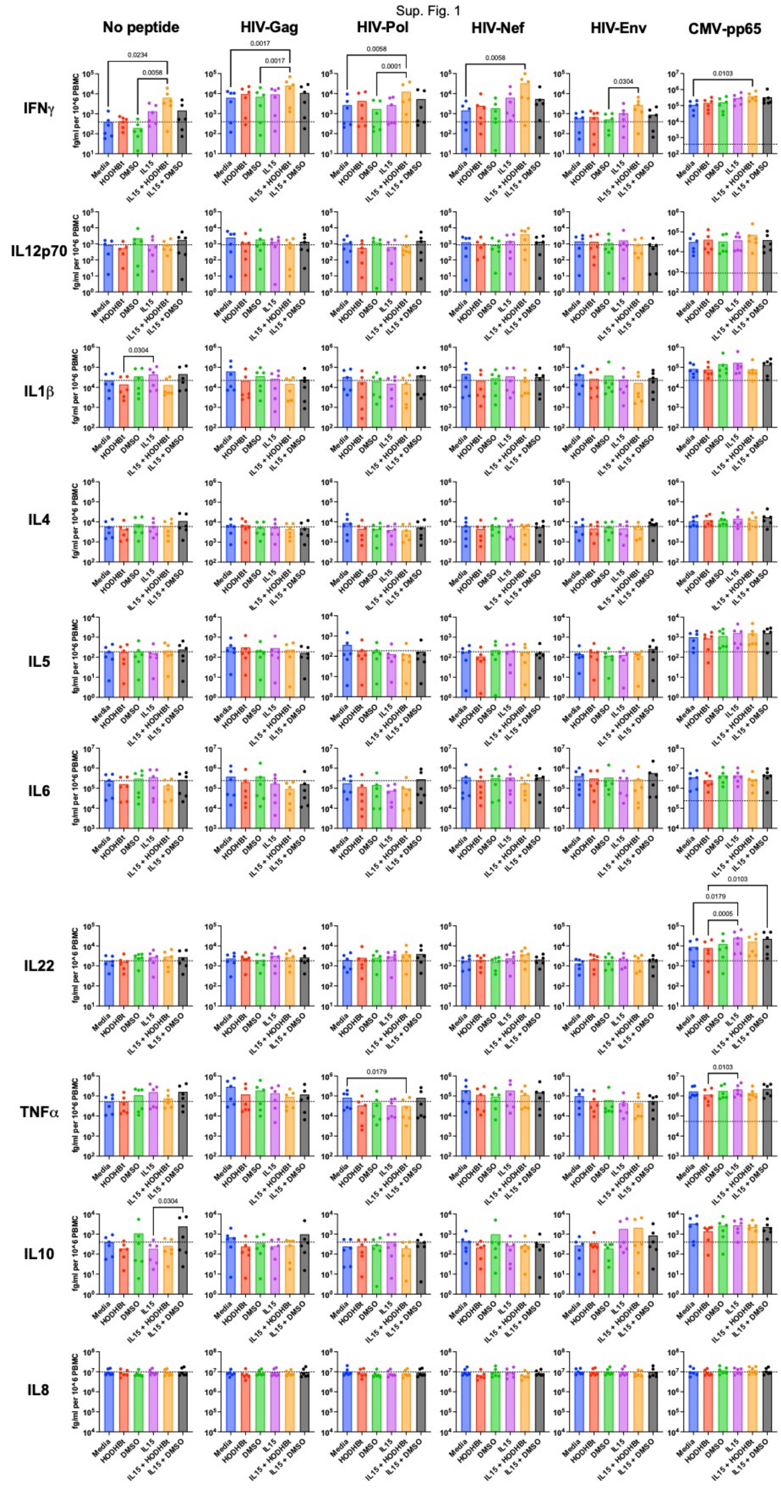

Supplementary Figure 1. Evaluation of cytokine secretion in the ELISPOT supernatants of 6 participants.

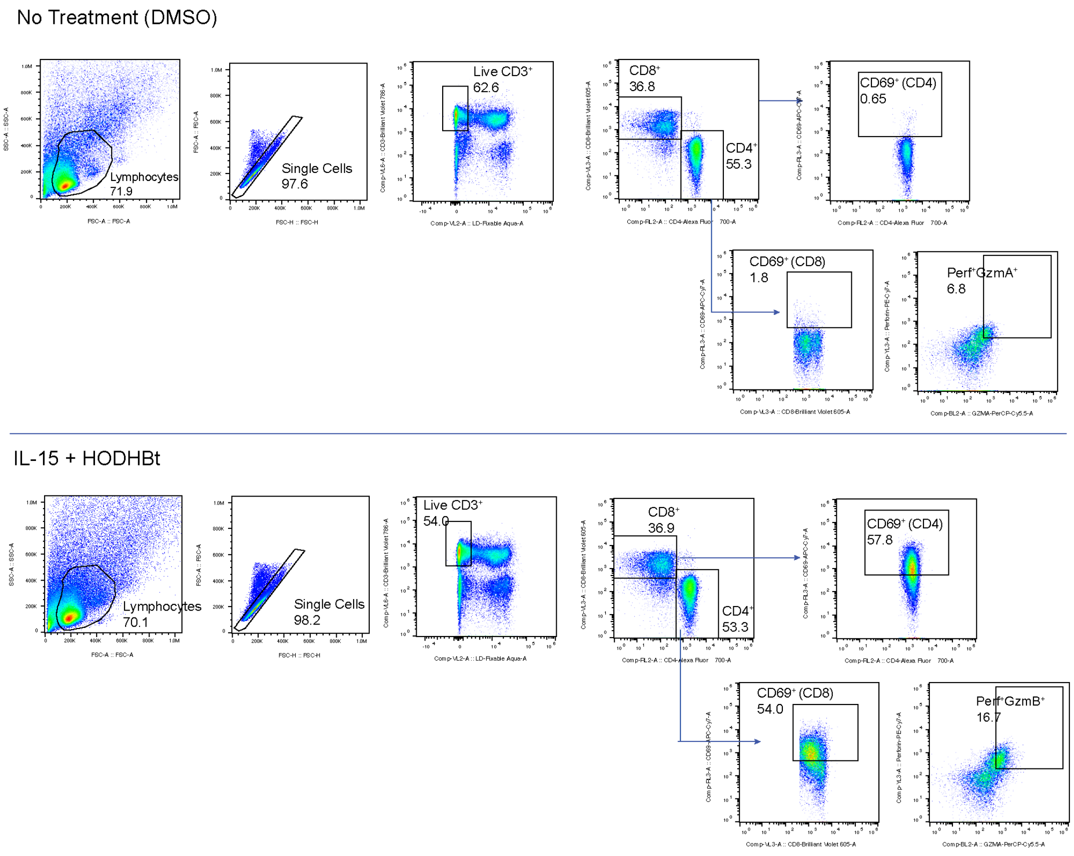

Supplementary Figure 2. Flow cytometry gating strategy used to assess HIV assays. Shown are day 3 results for donor OM5334.

Supplementary Table 1. Demographic and virologic parameters from A5321 study participants.

Participant ID	*Age at A5321 entry (years)*	*Sex*	*Race, Ethnicity*	*Years on ART at A5321 entry*	*Start date of initial ARV regimen*	*Year of ART initiation*	*Pre-ART CD4+ T-cell count (cells/mm³)*	*Pre-ART CD8+ T-cell count (cells/mm³)*	*A5321 entry CD4+ T-cell count (cells/mm³)*	*A5321 entry CD8+ T-cell count (cells/mm³)*	*A5321 entry ARV regimen*
**13820**	52	Male	Hispanic (Regardless of Race)	4.3	18-Jun-09	2009	261	1014.5	389	624	FTC TDF RAL
**111834**	46	Male	White Non-Hispanic	11.5	28-May-02	2002	97	1694.5	548	660	FTC TDF EFV
**112213**	46	Male	Hispanic (Regardless of Race)	4.2	16-Jul-09	2009	410.5	2066	799	1126	FTC TDF RTV DRV
**112222**	69	Female	White Non-Hispanic	4.4	9-Jul-09	2009	155	686.5	281	339	FTC TDF RAL
**141748**	45	Female	Black Non-Hispanic	7.4	6-Jun-06	2006	248.5	730	598	445	FTC TDF EFV
**214356**	31	Male	White Non-Hispanic	4.4	13-Jun-09	2009	322	580	760	336	FTC TDF RAL
**232326**	60	Female	White Non-Hispanic	14.8	18-Mar-99	1999	47	195.5	922	965	3TC ZDV EFV
**233303**	38	Male	White Non-Hispanic	7.1	27-Nov-06	2006	447	1829.5	699	853	FTC TDF EFV
**321348**	44	Male	White Non-Hispanic	7.4	6-Sep-06	2006	216.5	889	549	898	ABC 3TC EFV
**362446**	51	Female	Black Non-Hispanic	7.3	6-Sep-06	2006	232	800.5	860	810	FTC TDF EFV
**540827**	46	Female	Hispanic (Regardless of Race)	14.3	30-Jun-99	1999	524	1112	914	764	DDI 3TC ATV
**541654**	49	Female	Hispanic (Regardless of Race)	6.1	1-Oct-07	2007	304	688	718	1017	FTC TDF RTV ATV
**580740**	58	Male	White Non-Hispanic	7.5	11-Jul-06	2006	283.5	1555	808	892	FTC TDF RAL
**611097**	37	Female	Hispanic (Regardless of Race)	7.1	10-Nov-06	2006	332.5	636.5	892	531	FTC TDF RTV ATV

Supplementary Table 2. Demographic and virologic parameters from Maple Leaf Clinic participants.

Participant ID	*Age at Sample Collection (years)*	*Sex*	*Race, Ethnicity*	*Duration of undetectable viral Load (months)*	*ARV regimen*	*Viral Load (Copie/ml)*	*Estimated Time Between infection and ART (months)*
**OM5011**	46	M	White Non-Hispanic	133	3TC ABC DTG	<50	38
**OM5267**	28	M	White Non-Hispanic	91	3TC ABC RAL	<50	4
**OM5334**	34	M	White Non-Hispanic	63	TAF FTC EVG COBI RPV	<50	2

Supplemental Acknowledgments

We would like to acknowledge all the members of the A5321 team, including Evelyn Hogg, Rebecca LeBlanc, Christine Scello, David Palm, Monica Gandhi, Courtney Fletcher, Catherine Godfrey, Anthony Podany, Elias Halvas, Joan Dragavon, Jeymohan Joseph, Rose Lagattuta, Leyi Lin, Hannah Mar, Susan Pederson, Kevin Robertson, Leah Rubin, Serena Spudich, Bernadette Jarocki, Sean Avedissian, Ann Collier, Jonathan Li, Sharon Riddler, Aimee Willett, Charles Rinaldo Jr., and Jenny Nguyen.

Below is a listing of all of the above individuals and their affiliations.

Name	Affiliation
Evelyn Hogg	ACTG Network Coordinating Center Social & Scientific Systems<br>8757 Georgia Avenue, 12th Floor Silver Spring, MD 20910-3714
Rebecca LeBlanc	Frontier Science & Technology Research Foundation, Inc.<br>4033 Maple Road<br>Amherst, NY 14226

Christine Scello	Frontier Science & Technology Research Foundation, Inc.<br>Data Management Center<br>4033 Maple Road<br>Amherst, NY 14226-1056
David Palm	Chapel Hill CRS<br><br>P.O. Box 12161<br>Research Triangle Park, NC 27709
Monica Gandhi	University of California San Francisco Division of HIV/AIDS, Box 1352<br>405 Irving Street, 2nd Floor<br>San Francisco, CA 94122-1352
Courtney V. Fletcher	College of Pharmacy<br>University of Nebraska Medical Center 986000 Nebraska Medical Center Omaha, NE 68198-6000
Catherine Godfrey	HIV Research Branch TRP/DAIDS/NIAID/NIH<br><br>5601 Fishers Lane Room 9E49 MSC 9830<br>Bethesda, MD 20852-9830
Anthony Podany	Antiviral Pharmacology Laboratory College of Pharmacy, RM 4007 University of Nebraska Medical Center 986045 Nebraska Medical Center Omaha, NE 68198-6045
Elias Halvas	University of Pittsburgh Virology Support Laboratory<br>Division of Infectious Diseases S813 Scaife Hall<br>3550 Terrace Street<br>Pittsburgh, PA 15261
Joan Dragavon	Virology Specialty Laboratory University of Washington<br>Research & Training Building<br>300 9th Avenue, Room 725<br>Seattle WA 98104-2499

Jeymohan Joseph	HIV Neuropathogenesis and Treatment Branch<br>National Institute of Mental Health Room 6219, MSC 9619<br>6001 Executive Boulevard<br>Bethesda, MD 20892-9619
Rose Lagattuta	11075 Santa Monica Boulevard, Ste. 200 Los Angeles, CA 90025
Leyi Lin	5601 Fishers Lane<br><br>MSC 9830, Room 9E47 Rockville, MD 20852
Hanna Mar	Statistical and Data Analysis Center Harvard School of Public Health FXB 643A<br>Boston, MA 02115
Susan Pedersen	University of North Carolina at Chapel Hill Bioinformatics Building, Suite 2100<br>130 Mason Farm Road<br>Chapel Hill, NC 27599-7215
Kevin Robertson	Chapel Hill CRS<br>Department of Neurology<br>2128 Physician Office Building 170 Manning Drive<br><br>Chapel Hill, NC 27599-7025
Leah H. Rubin	Department of Neurology Johns Hopkins University 600 N. Wolfe Street<br>Meyer 6-113a<br>Baltimore MD 21287
Serena S. Spudich	Cornell CRS<br>Yale University<br>PO Box 208018

	15 York Street<br>New Haven, CT 06520
Bernadette Jarocki	Frontier Science & Technology Research Foundation, Inc.<br>4033 Maple Road<br>Amherst, NY 14226-1056
Sean Avedissian	University of Nebraska Medical Center 42nd and Emile<br>Omaha NE 68198
Ann Collier	University of Washington School of Medicine<br>Harborview Medical Center<br>Box 359929<br>325 9th Avenue<br>Seattle, WA 98104
Jonathan Li	Brigham and Women’s Hospital 65 Landsdowne Street, Rm 421 Cambridge, MA 02139
Sharon Riddler	University of Pittsburgh CRS 3520 Fifth Avenue<br>Keystone Building, Suite 510 Pittsburgh, PA 15213-2582
Aimee Willett	Frontier Science & Technology Research Foundation, Inc. 4033 Maple Road Amherst, NY 4226-1056
Charles Rinaldo Jr.	Infectious Diseases & Microbiology University of Pittsburgh<br>A419 Crabtree Hall 130 DeSoto Street Pittsburgh, PA 15261<br>Phone: 412-624-3928
Jenny Nguyen	Laboratory Science Group ACTG Network Coordinating Center Social & Scientific Systems, Inc.8757 Georgia Avenue, 12th Floor Silver Spring, MD 20910
